# Hæmophilia

**Published:** 1900-05

**Authors:** Charles A. Porter

**Affiliations:** Boston, Mass.


### HÆMOPHILIA.[1]

BY CHARLES A. PORTER, M.D., BOSTON, MASS.

In connection with the case which Professor Fillebrown reports to-night, he has asked me to read to you a short paper on the general surgical aspects of hæmophilia, a disease which interests alike the dentist and the general surgeon.

Fortunately this disease is a relatively rare affection, but, as in tetanus, the appallingly fatal results which may follow trivial wounds make it wise for us all to understand something of the general symptomatology of the disease, and to know at least where further knowledge may be acquired on occasion.

In the literature scattered cases are found, dating back as far as the eleventh or twelfth century, where excessive or fatal hemorrhages followed slight wounds; but it was not until 1828, when Schönlein collected and described a number of cases, that the dis-

[1] Read before the American Academy of Dental Science, December 6, 1899.

ease recived its present name. Three men in particular have written monographs on the subject,—Grandidier, Immermann, and J. Wickham Legg. His last article, in Albrett's "System of Medicine," is the most recent and comprehensive description of the disease which I can find. Early in the present century several shorter papers appeared from American authors, and the popular term, "bleeder," first arose on this side of the water.

Legg defines hæmophilia as "a disease congenital and hereditary, marked by a tendency to immoderate bleeding on slight causes, lasting generally through the life of the patient, and further accompanied by joint-affections, which are often as wearisome to the patient as the tendency to external hemorrhages is dangerous." From this definition it can be seen that hæmophilia proper means much more than a single severe hemorrhage from slight cause, and has nothing to do with bleedings in anæmia, Bright's disease, scurvy, or jaundice, and is different from the hemorrhages in the new-born which do not tend to recur in later life.

It can be stated with truth that hæmophilia is, of all diseases, the most hereditary; *e.g.*, in Tenna, in Switzerland, the affection has beeen traced back in two families for three hundred years. In the Appleton-Swain families, in Reading, Mass., cases have occurred for nearly two hundred years; and F. F. Brown is quoted by Osler as knowing of a case in the seventh generation. Several cases have been reported where all the male children have died; as bleeders are remarkably prolific, this usually means a large mortality. In one family all the male children died before reaching the age of ten.

Furthermore, the mode of transmission is extremely interesting. This disease affects males much more frequently than females, in the proportion of twelve to one; yet it is through the female line that the disease is almost always transmitted: in a given family the sons are bleeders, the daughters generally healthy; in the next generation the sons of the daughters are bleeders, while the children of the male bleeder are free. Not all the children, as a rule, are affected, but two or three brothers may suffer.

While by the older writers the Anglo-Germanic races and the Jews were thought to be peculiarly susceptible, it now seems doubtful whether race plays a very important part. Recently cases are reported among the natives of India and Japan.

The time of life at which the hemorrhages take place is variable: rarely in fœtal life unusual blood extravasations are reported; in the first few days bleeding is more frequent, and ritual circumcision among the Jews, celebrated on the eighth day, has often proved fatal in hæmophilic children. It is interesting to note that the women in bleeder families, contrary to what might be expected, rarely suffer from profuse menstruation or unusual loss of blood at childbirth.

The hemorrhages in hæmophilia may be spontaneous or traumatic; from the mucous membranes; into the skin or subcutaneous tissues; into the joint cavities; from the internal organs, as hæmaturia of renal origin; and finally, from any wound, ulcer, or abrasion of the skin, however trivial,—a pin prick, cutting the frænum of the tongue or penis, boring the ears. In childhood, epistaxis is the most common manifestation; next come large ecchymoses and subcutaneous hæmatomata, with or without preceding injury. In later life, hemorrhages from the internal organs, and especially recurrent hemorrhages in the joints or periarticular tissues.

The periods especially liable to hemorrhage are the first and second dentitions and puberty. In the great majority of cases some manifestation of the inherited disease has occurred before the fifth year. Grandidier reports one hundred and fifty-two cases in boy bleeders, where eighty-one (more than fifty per cent.) died before the eighth year. Patients rarely die from the first hemorrhage. At or about twenty-one is another danger period. When middle life is passed the disease tends to abate, and rarely the diathesis seems to be completely outgrown.

This tendency to hemorrhage is undoubtedly variable in a bleeder: a given injury at one time will cause severe loss of blood; at another a similar wound behaves as it would in a normal individual. Damp seasons, exposure to cold, nervous excitement, are factors often spoken of, influences, however, which are always open to question.

Probably these joint-affections have erroneously led to the conclusion that this disease was related to rheumatism, or purpura rheumatica. More than once knees have been excised by surgeons, with fatal results, under the belief that the joint lesion was tubercular.

Of especial interest to you, of course, are the frequent hemorrhages after operations in the mouth, especially after the extraction of teeth; and tooth-pulling in bleeder families has always been a procedure which the dentist has wished to relegate to his confrères. This trivial operation was four times fatal in one family, and by most authorities is considered unjustifiable except under very peculiar circumstances. The opening of alveolar abscesses has proved more dangerous than allowing them to rupture spontaneously. While it is manifestly impossible for busy dentists or surgeons to inquire in out-patient clinics in regard to hæmophilia, it is certainly obligatory that every physician who has treated a bleeder should carefully explain to the patient or his family the grave danger he runs from any future operation.

The points in the past history of a suspected case to be inquired about particularly are excessive hemorrhage from trivial wounds,—lancing the gums, nose-bleed, large recurrent ecchymoses, so-called rheumatism in the joints without fever, hemorrhages from internal organs. A family history of bleeding will, of course, be of great value.

The amount and rapidity of hemorrhage in these cases varies considerably. A sharp loss of blood may immediately follow the extraction of a tooth, which rapidly exsanguinates the patient; or the blood may clot normally at first and the bleeding cease, to be followed by a continuous slight ooze in spite of the usual treatment, which at the end of days, or even weeks, reduces the patient to a condition of severe anæmia, or proves fatal. From this condition patients not infrequently slowly recover just as their life is despaired of; indeed, some physicians believe that true cases of hæmophilia rarely stop bleeding until almost dead, and, having in other cases tried in vain the usual remedies, calmly await this period.

With regard to the pathology of the disease very little is known. In the majority of autopsies nothing unusual has been found either in the blood or tissues. Some investigators speak of abnormally small arteries, thinning of the middle coat, fatty degeneration of the intima and media. These later changes can be satisfactorily explained by the severe anæmia before death. Some find in fresh cases an increased number of white cells; others nothing abnormal. The leucocytosis in late cases is that which accompanies all grave hemorrhages. Wright, in certain cases, has found that the blood coagulated very slowly, and attributes to this the continuous flow of blood, theoretically due to deficiency of the lime salts or nucleoalbumins. In support of this theory, some bleeder children have undoubtedly an abnormal craving for plaster.

That this delayed coagulability is not always present is shown by the fact that not infrequently a normal clot is formed; yet this is later washed away by the capillary ooze about it. It seems, therefore, that the real lesion must be situated in the capillaries or small arterioles, for here normal thrombosis does not occur, though the blood whch flows from the wound may clot in the vessel. Patients with hæmophilia are apt to be very thin skinned, neurasthenic, liable to sudden flushings and vasomotor disturbances; it may be that some hereditary deficiency exists which interferes with the action of the vasoconstrictors.

With such an indefinite, irregular, or unknown pathology, it is evident that our treatment must be chiefly empirical, yet always directed towards the formation of a clot *in the vessel walls.* The multiplicity of remedies suggested and tried speaks for their frequent uselessness. I shall speak, therefore, only of those which have been found successful, or at least rest on some rational basis. The female members of a bleeder family assume a serious risk for their offspring by marriage. Bleeder children should be protected in every way from injury.

Whether iron and other tonics, particularly sulphate of soda, as advocated by Legg, much affect the disease is open to question, but those physicians who have had charge of bleeder families feel sure that bland, unirritating diet, frequent catharsis, and a quiet, unemotional life, with change of climate, have favorably influenced the recurrence of hemorrhage. What interests us most, however, are the measures which may be taken to stop an actual hemorrhage. It is difficult to gather from the literature a definite opinion as to the real efficacy of the ordinary styptics given internally or applied locally, for after the administration of certain drugs it is often hard to determine whether the hæmophilia ceased because of their administration, or spontaneously. Ergot seems inefficient. It seems also doubtful if iron, given internally, can effect the coagulability of the blood. The two drugs which are spoken of most favorably are hydrastis canadensis, in doses of thirty minims every four hours, especially valuable in spontaneous hemorrhage from internal organs, and dilute sulphuric acid, twenty minims in an ounce of water four times a day. Sidal used the latter drug successfully in three cases, and stopped the hemorrhage in forty-eight hours, whereas the cases had previously bled to exsanguination. Perhaps the administration of calcium chloride is the most scientific treatment. Wright and J. Clifford Perry both report cases where this drug was used with wonderful success: one a man of twenty, alveolar abcess, incision one-eighth of an inch long, profuse hemorrhage in spite of pressure and styptics. He had several times bled until he fainted. Two brothers had died in infancy after trivial wounds. Calcium chloride was given in grain doses every two hours, and after three doses the blood formed a firm clot. Wright reports one case in which the coagulation time exceeded fifty-four minutes. After two drachms of calcium chloride had been given at two-hourly intervals, the coagulation time was reduced to twenty-five minutes, and after two more doses, to thirteen and one-half minutes. In a normal case the coagulation time was reduced, after a few doses, from fourteen minutes to six and three-fourths minutes. This drug is therefore worthy of trial. But in Dr. Fillebrown's case, and several others, no benefit resulted.

Inhalations of carbon dioxide gas have been successful; so, too, continuous inhalations of oxygen. Though the dangers and difficulties of transfusion of human blood are very great, I see no reason why it should not be done as a last resort. Fry in the New York *Medical Record* of July 3, 1898, reports three very interesting and successful cases where he infused normal horse serum. One patient received in all three hundred cubic centimetres; the improvement was remarkable, hemorrhage ceased, and no harm resulted from these injections.

Whichever of these general measures the surgeon chooses to adopt, there is no doubt that absolute quiet should be insured to the patient. Davies, who has had marked success with two hæmophilic families, placed the patient, immediately on the occurrence of hemorrhage, in a dark, quiet room, forbidding conversation, and withholding food entirely for two days. The hunger is relieved by small doses of opium, the thirst by a little ice-water. He has had no success with any general or local styptics whatever.

The local measures may be divided into pressure and styptics. It seems of great importance that before pressure is applied the cavity should be thoroughly cleared of clots. Finger pressure is most intelligent, but has usually to be supplemented by whatever mechanical device the dental surgeon can devise. The best local styptic is gauze dipped in perchloride of iron and firmly packed into the cavity. The jaws may be held together with elastic bands or an interdental plate or splint fitted. A wooden or cork peg has been driven through the gauze into the alveolus with success, but unfortunately oozing often occurs subsequently from the ulcerated gum. There are those who believe that the frequent traumata in connection with packings induce fresh hemorrhage, and do more harm than good. Antipyrin solution, twenty or thirty grains to the ounce, applied on absorbent cotton, is a powerful styptic. The actual cautery, which often temporarily arrests the bleeding, rarely is of permanent benefit. Hydrogen peroxide is a valuable styptic. Davies, whom I have quoted before, has had remarkable success with ethyl chloride. Immediately after the extraction of the tooth he freezes the clot with a spray, and maintains this condition for several minutes. In many cases he has not failed to arrest the hemorrhage. Normal human blood has been poured into the wounds of hæmophilic patients, and successes have been reported. General cardiac stimulants, unless absolutely demanded, are definitely contraindicated, especially alcohol. It is hard to see of what use salt infusion can be, for the previously diluted blood would be rendered thereby more incoagulable.

These are the measures which appear most useful:

1. Absolute quiet.

2. No food for two days.

3. Opium in small doses.

4. Internally: (1) Dilute sulphuric acid; (2) hydrastis canadensis; (3) calcium chloride.

5. Serum infusion or direct blood transfusion only as a last resort.

6. Locally: (1) Cleaning out the clot; (2) freezing with ethyl chloride; (3) antipyrin or peroxide; (4) normal blood for clotting.

7. And finally. pressure evenly and firmly applied by whatever device will cause least disturbance or sloughing.

If in spite of these measures, carefully and thoroughly carried out, the patient dies, the surgeon has done all in his power, and as I have said, most cases come near to death before the hemorrhage ceases.

